# WDR83/MORG1 inhibits RRAG GTPase-MTORC1 signaling to facilitate basal autophagy

**DOI:** 10.1080/15548627.2024.2322457

**Published:** 2024-03-07

**Authors:** Athanasios Kournoutis, Trond Lamark, Terje Johansen, Yakubu Princely Abudu

**Affiliations:** a Autophagy Research Group, Department of Medical Biology, University of Tromsø-The Arctic University of Norway, Tromsø, Norway; b Nanoscopy Group, Department of Physics and Technology, University of Tromsø-The Arctic University of Norway, Tromsø, Norway

**Keywords:** Autophagy, breast cancer, MORG1, MTORC1, RRAG GTPases, SQSTM1/p62

## Abstract

Macroautophagy/autophagy is a conserved lysosomal degradation process composed of both selective and nonselective degradation pathways. The latter occurs upon nutrient depletion. Selective autophagy exerts quality control of damaged organelles and macromolecules and is going on also under nutrient-replete conditions. Proper regulation of autophagy is vital for cellular homeostasis and prevention of disease. During nutrient availability, autophagy is inhibited by the MTORC1 signaling pathway. However, selective, basal autophagy occurs continuously. How the MTORC1 pathway is fine-tuned to facilitate basal constitutive autophagy is unclear. Recently, we identified the WD-domain repeat protein WDR83/MORG1 as a negative regulator of MTORC1 signaling allowing basal, selective autophagy. WDR83 interacts with both the Ragulator and active RRAG GTPases to prevent recruitment of the MTORC1 complex to the lysosome. Consequently, WDR83 depletion leads to hyperactivation of the MTORC1 pathway and a strong decrease in basal autophagy. As a consequence of WDR83 depletion cell proliferation and migration increase and low levels of *WDR83* mRNA are correlated with poor prognosis for several cancers.

Autophagy, an important cellular recycling and quality control system is tightly regulated to maintain cellular homeostasis and prevent diseases. Autophagy ensures the prompt degradation and recycling of surplus, worn-out or dangerous cellular components. Under normal conditions, autophagy occurs constitutively but increases substantially upon stress such as starvation and infection. How autophagy is regulated under different stress conditions is not entirely clear. The MTOR (mechanistic target of rapamycin kinase) complex 1 (MTORC1) pathway regulates autophagy under conditions of nutrient availability and scarcity. When nutrients are available, the MTORC1 pathway inhibits autophagy by controlling several aspects of the autophagy process including initiation, maturation and lysosomal biogenesis. In the presence of nutrients, MTORC1, comprising the kinase MTOR and several associated proteins, is recruited to the lysosome by the RRAG GTPases, which are themselves anchored to the lysosome by the Ragulator complex. At the lysosome, MTOR is activated by the small GTPase RHEB to promote protein synthesis and inhibit degradation. However, when nutrients become scarce, MTORC1 is inhibited allowing the initiation of autophagy. Interestingly, how MTORC1 is regulated during conditions of nutrient availability to allow for constitutive basal autophagy is not entirely clear. In a recent study, we identified the WD-domain repeat protein WDR83/MORG1 (WD repeat domain 83) as a negative regulator of the MTORC1 signaling pathway [1]. WDR83 fine tunes MTORC1 signaling during conditions of nutrient availability to facilitate basal constitutive autophagy flux.

Initially, we identified WDR83 as an interaction partner of SQSTM1/p62 from immunoprecipitation of SQSTM1 from both MEF and HEK293 cells, but WDR83 is not degraded by autophagy. However, *WDR83* knockout results in accumulation of selective autophagy receptors and the human Atg8-family proteins suggesting inhibition of basal autophagy flux. We discovered that WDR83 localizes to the lysosome as it colocalizes with LAMP1, but the effect of WDR83 on basal autophagy was not from a direct effect on the lysosomal function. By combining standard immunoprecipitation and APEX2-based proximity ligation with quantitative proteomics we could identify interacting and proximity partners of WDR83. We consistently identified several components of the MTORC1 signaling pathways as putative interacting or proximity partners of WDR83, including MTOR, RPTOR/raptor, the RRAG GTPases and Ragulator complex proteins. Furthermore, one of the earliest known partners of WDR83 is the Ragulator complex protein LAMTOR3/MP1.

We then discovered that knockout of *WDR83* leads to hyperactivation of the MTORC1 signaling pathway. Key substrates of MTORC1, including RPS6KB1/S6K1 (ribosomal protein S6 kinase B1), EIF4EBP1 (eukaryotic translation initiation factor 4E binding protein 1), lysosomal transcription factors TFEB and TFE3 and the autophagy initiation complex protein ULK1, are significantly phosphorylated in the absence of WDR83. This suggested that the inhibition of basal autophagy in *WDR83* knockout cells is a result of MTORC1 hyperactivation indicating a negative effect of WDR83 on MTORC1. To understand mechanistically how WDR83 regulates MTORC1, we asked if WDR83 can directly interact with components of the MTORC1 signaling pathway. Using several interaction assays, we found that WDR83 directly interacts with the Ragulator complex proteins and the active RRAG GTPases and modulates the Ragulator-dependent recruitment of the RRAG GTPases to the lysosome. In the presence of WDR83, the interaction between the Ragulator complex proteins and RRAG GTPases is significantly reduced whereas, this interaction increases in the absence of WDR83. Using live cell confocal microscopy, we discovered that knockout of *WDR83* leads to increased colocalization between MTOR and LAMP1, whereas overexpression of WDR83 reduces the localization of MTOR to the lysosome. Furthermore, using lysosomal immunoprecipitation (LysoIP), we discovered that knockout of *WDR83* leads to an increased recruitment of MTORC1 complex proteins including MTOR, RPTOR and RRAG GTPases to the lysosome, whereas the amount of the Ragulator complex proteins on the lysosome does not change. These results suggested that the interaction of WDR83 with both the Ragulator complex and the active RRAG GTPases regulates the spatiotemporal localization of MTORC1 to the lysosome. This regulation fine tunes the activity of MTORC1 to facilitate basal autophagy during conditions of nutrient availability (Figure 1).

**Figure 1.**
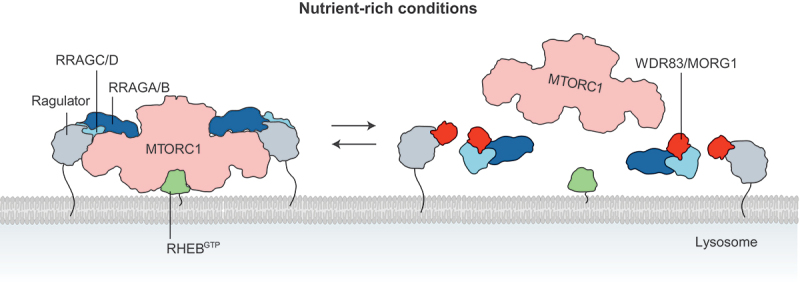
WDR83 negatively regulates MTORC1 signaling. WDR83 (red) interacts with both the ragulator complex (gray) and the RRAG GTPases (light and dark blue) to restrict the lysosomal recruitment of the RRAG GTPases by the ragulator complex under nutrient-rich conditions. This restriction fine tunes the spatiotemporal localization of MTORC1 (salmon red) to the lysosomal surface for activation by the small GTPase RHEB (green). The MTORC1 depicted here consists of MTOR, RPTOR, DEPTOR, MLST8 and AKT1S1/PRAS40.

We also identified WDR83 as an interaction partner of SQSTM1, but our data do not support a direct role for this interaction in WDR83-mediated regulation of MTORC1 signaling in basal, nutrient-replete conditions. Our data revealed a competitive interaction of WDR83 and SQSTM1 with the RRAG GTPases. In the presence of WDR83, the interaction between SQSTM1 and the RRAG GTPases is significantly reduced. SQSTM1 is a positive regulator of MTORC1 signaling in response to leucine stimulation following amino acid starvation. This depends on the interaction of SQSTM1 with the RRAG GTPases. Our data confirmed that *SQSTM1* knockout significantly reduces MTORC1 signaling in response to leucine stimulation following amino acid starvation. However, while our data confirmed the importance of SQSTM1 upon amino acid withdrawal and restimulation, we found that *SQSTM1* knockout does not affect MTORC1 signaling under basal conditions nor the interaction between RRAG GTPases and WDR83. While *WDR83* knockout significantly upregulates the interaction between RRAG GTPases and RPTOR, *SQSTM1* knockout has no effect on this interaction. Hence, under basal conditions, WDR83 modulates MTORC1 activity to facilitate basal autophagy flux, while SQSTM1 has no effect. However, SQSTM1 is vital to MTORC1 signaling during amino acid withdrawal and restimulation. Interestingly, under basal condition, we found that WDR83 localizes extensively to the lysosome and only few lysosomes are positive for SQSTM1 puncta, whereas localization of SQSTM1 to the lysosome increases upon amino acid withdrawal and restimulation. It appears that SQSTM1 regulates MTORC1 signaling during recovery from amino acid starvation which corresponds to the role of SQSTM1 as a stress response protein.

Other proteins such as SH3BP4 (SH3 domain binding protein 4) and CDKN1B/p27 also regulate MTORC1 signaling through interaction with either the RRAG GTPases or Ragulator complex. In conclusion, our work shows that WDR83 is an important regulator of the MTORC1-autophagy-lysosomal pathway, modulating the activity of MTORC1 during conditions of amino acid availability and scarcity.
